# Biofilm Formation in Streptococcus agalactiae Is Inhibited by a Small Regulatory RNA Regulated by the Two-Component System CiaRH

**DOI:** 10.1128/spectrum.00635-22

**Published:** 2022-08-18

**Authors:** Nancy Jabbour, Eric Morello, Emilie Camiade, Marie-Frédérique Lartigue

**Affiliations:** a ISP, UMR1282, Université de Tours, INRAE, Tours, France; b CHRU de Tours, Service de Bactériologie-Virologie-Hygiène, Tours, France; Ohio State University

**Keywords:** *Streptococcus agalactiae*, CiaRH, sRNAs, csRNAs, regulation, adaptation, biofilm, SAP pullulanase

## Abstract

Regulatory small RNAs (sRNAs) are involved in the adaptation of bacteria to their environment. CiaR-dependent sRNAs (csRNAs) are controlled by the regulatory two-component system (TCS) CiaRH, which is widely conserved in streptococci. Except for Streptococcus pneumoniae and Streptococcus sanguinis, the targets of these csRNAs have not yet been investigated. Streptococcus agalactiae, the leading cause of neonatal infections, has four conserved csRNA genes, namely, *srn015*, *srn024*, *srn070*, and *srn085*. Here, we demonstrate the importance of the direct repeat TTTAAG-N5-TTTAAG in the regulation of these csRNAs by CiaRH. A 24-nucleotide Srn024-*sap* RNA base-pairing region is predicted *in silico*. The *sap* gene encodes a LPXTG-cell wall-anchored pullulanase. This protein cleaves α-glucan polysaccharides such as pullulan and glycogen present in the environment to release glucose and is involved in adhesion to human cervical epithelial cells. Inactivation of S. agalactiae pullulanase (SAP) leads to no bacterial growth in a medium with only pullulan as a carbon source and reduced biofilm formation, while deletion of *ciaRH* and *srn024* genes significantly increases bacterial growth and biofilm formation. Using a new translational fusion vector, we demonstrated that Srn024 is involved in the posttranscriptional regulation of *sap* expression. Complementary base pair exchanges in S. agalactiae suggest that Srn024 interacts directly with *sap* mRNA and that disruption of this RNA pairing is sufficient to yield the biofilm phenotype of Srn024 deletion. These results suggest the involvement of Srn024 in the adaptation of S. agalactiae to environmental changes and biofilm formation, likely through the regulation of the *sap* gene.

**IMPORTANCE** Although Streptococcus agalactiae is a commensal bacterium of the human digestive and genitourinary tracts, it is also an opportunistic pathogen for humans and other animals. As the main cause of neonatal infections, it is responsible for pneumonia, bacteremia, and meningitis. However, its adaptation to these different ecological niches is not fully understood. Bacterial regulatory networks are involved in this adaptation, and the regulatory TCSs (e.g., CiaRH), as well as the regulatory sRNAs, are part of it. This study is the first step to understand the role of csRNAs in the adaptation of S. agalactiae. This bacterium does not currently exhibit extensive antibiotic resistance. However, it is crucial to find alternatives before multidrug resistance emerges. Therefore, we propose that drugs targeting regulatory RNAs with Srn024-like activities would affect pathogens by reducing their abilities to form biofilm and to adapt to host niches.

## INTRODUCTION

Streptococcus agalactiae, a group B streptococcus, is a chain-forming Gram-positive opportunistic pathogen. It was first associated with bovine mastitis and is also known to cause outbreaks in fish farms (1, 2). S. agalactiae colonizes asymptomatically the digestive and genitourinary tracts of 10% to 30% of humans (3). It is the leading cause of invasive infections in neonates, causing pneumonia, bacteremia, and meningitis mainly via maternal transmission, and has emerged as an increasing cause of invasive diseases in immunocompromised and elderly adults (4, 5). The capacity of S. agalactiae to colonize and survive in such different environments underlines its ability to adapt to encountered changes. A first line of control is transcriptional, with regulators such as two-component systems (TCSs) that respond to physical and chemical perturbations. It is followed by posttranscriptional regulations in which regulatory small RNAs (sRNAs) may affect RNA elongation, processing/degradation, and translation (6).

The TCS CiaRH in Streptococcus pneumoniae regulates the transcription of sRNAs (CiaR-dependent sRNAs [csRNAs]) through the binding of CiaR protein to a direct repeat, TTTAAG-N5-TTTAAG (7). A previous study showed that S. pneumoniae csRNA5 was involved in lung infection and thus in adaptation to its environment (8). In S. agalactiae, several studies described sRNAs (9–13). Among them, four are potential csRNAs. They were first predicted *in silico* and named csRNA10, csRNA11, csRNA12, and csRNA14 (10). Then, they were detected by high-throughput RNA sequencing (RNA-seq) and renamed Srn015, Srn024, Srn070, and Srn085, respectively, and the expression of three of them was confirmed by Northern blotting in S. agalactiae strain NEM316 (11).

Here, we examined the role of csRNAs in S. agalactiae adaptation to its environment. Our results showed that csRNAs are conserved in S. agalactiae and controlled by the TCS CiaRH. We demonstrated that Srn024 is involved in *sap* posttranscriptional regulation. The *sap* gene encodes an LPXTG-cell wall-anchored pullulanase that cleaves α-glucan polysaccharides such as pullulan and glycogen found in the environment to release glucose, a nutrient source for bacteria (14). In S. agalactiae, S. agalactiae pullulanase (SAP) is known to bind to human cervical epithelial cells (15). Adhesion to matrix constitutes a critical step to promote biofilm formation and contributes to bacterium virulence. We demonstrated (i) that inactivation of SAP leads to no bacterial growth in a medium with only pullulan as a carbon source, while deletion of *ciaRH* and *srn024* genes significantly increases bacterial growth versus the wild-type (WT) strain, and (ii) that SAP induces biofilm formation, whereas CiaRH and Srn024 repress biofilm production of S. agalactiae. The regulatory network of CiaRH, Srn024, and SAP could thus be involved in the host colonization. We propose that drugs targeting regulatory RNAs with Srn024-like activities would affect pathogens by reducing their ability to produce biofilm and to adapt to host niches.

## RESULTS

### csRNAs are conserved in S. agalactiae.

We reported the identification of 47 putative *trans*-acting sRNAs in S. agalactiae strain NEM316 (11). Among them, the four csRNAs (Srn015, Srn024, Srn070, and Srn085, which are 140, 90, 112, and 218 nucleotides [nt] long, respectively) were expressed. Their corresponding genes, namely, *srn015*, *srn024*, *srn070*, and *srn085*, are located between *pgi* and *gbs0438*, between *gbs0600* and *gbs0601*, between *gbs1697* and *gbs1698*, and between *gbs2087* and *gbs2088*, respectively. Sequence homology searches using all available S. agalactiae shotgun and complete genome sequences reveal that *srn015*, *srn024*, s*rn070*, and *srn085* are conserved in S. agalactiae, with a low degree of polymorphism (>98% nucleotide identity). Thus, csRNAs belong to S. agalactiae regulatory RNA core genes. Interestingly, BLAST analysis did not result in significant matches when performed in other streptococcal genomes, suggesting that *srn015*, *srn024*, *srn070*, and *srn085* are specific to S. agalactiae. The csRNA conservation within S. agalactiae suggests that they have an important role in bacterial physiology.

### csRNAs are regulated by the TCS CiaRH.

**FIG 1:**
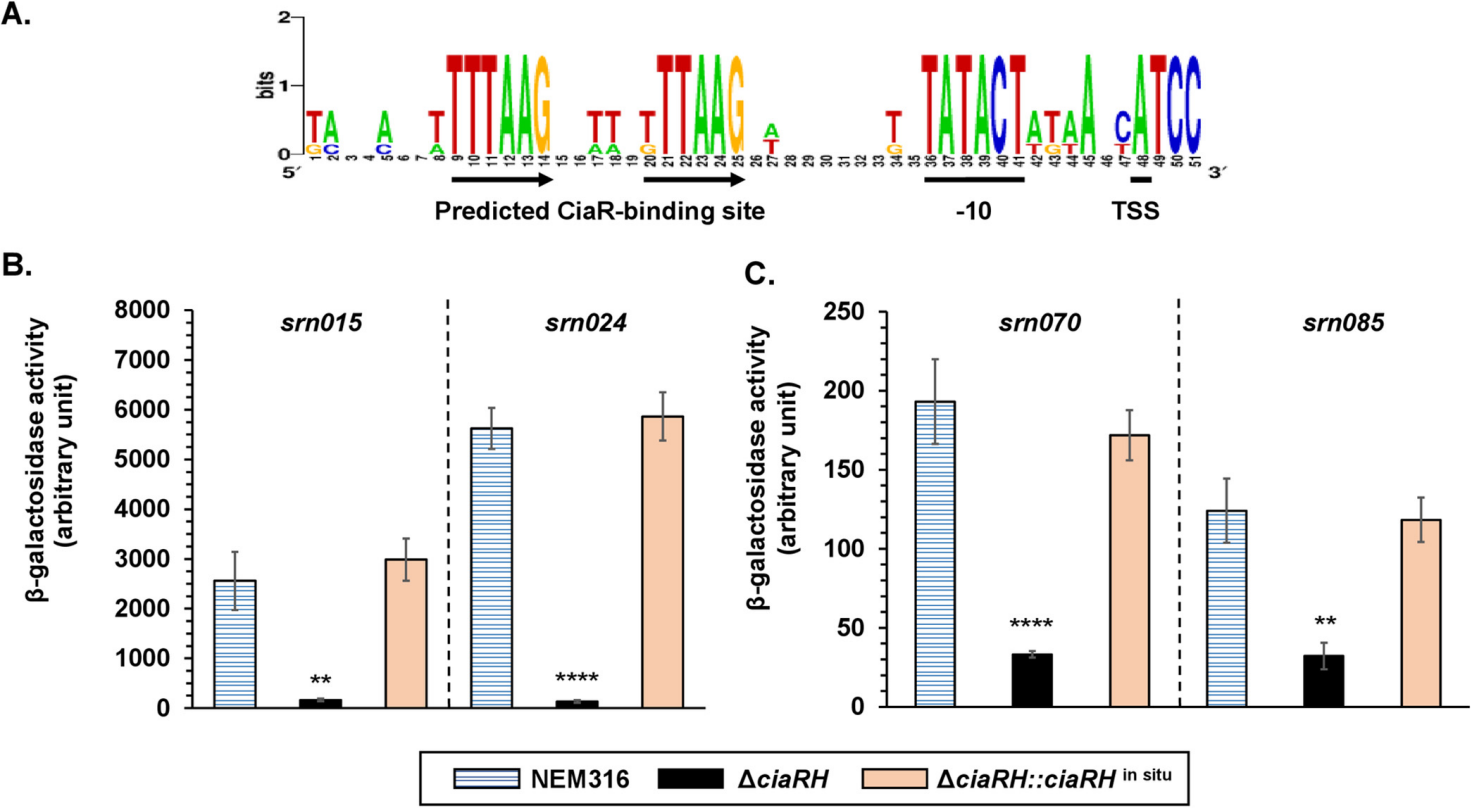
Activation of the four csRNA promoters by the TCS CiaRH. (A) Sequence logo of S. agalactiae promoters for csRNA predicted genes. The CiaR-related repeats are underlined by arrows. The transcriptional start site and the −10 region are underlined. WebLogo produced the logo. (B and C) The promoter activities of *srn015* and *srn024* (B) and *srn070* and *srn085* (C) were measured in S. agalactiae NEM316, Δ*ciaRH*, and Δ*ciaRH*::*ciaRH^in situ^* strains. Results are presented as means ± standard deviations of three independent experiments. The significance was determined by ANOVAs and Student *t* tests. **, *P < *0.01; ****, *P < *0.0001.

The presence of a conserved motif, TTTAAG-N5-TTTAAG, was detected within the promoter regions of Srn015, Srn024, Srn070, and Srn085 using WebLogo (Fig. 1A) (16). This sequence was previously identified as a CiaR binding site in S. pneumoniae (7). This result suggested CiaRH regulation of these csRNAs. To test this possibility, a strain in which the *ciaRH* operon was inactivated (Δ*ciaRH*) was constructed along with the corresponding complemented strain (Δ*ciaRH*::*ciaRH^in situ^*). The promoter regions of each of the four csRNA genes were cloned upstream of the *lacZ* gene (encoding β-galactosidase enzyme) into the promoter probe vector pTCV-*lacZ*. The resulting plasmids were introduced into NEM316 WT, Δ*ciaRH*, and Δ*ciaRH*::*ciaRH^in situ^* strains, and the β-galactosidase activity of each strain was determined at the mid-exponential growth phase in Todd-Hewitt (TH) broth. The four tested promoters were active in the NEM316 and Δ*ciaRH*::*ciaRH^in situ^* strains but had strongly reduced activity in the Δ*ciaRH* strain (Fig. 1B and C). Moreover, an important difference was observed in the promoter activities of the four csRNAs. Indeed, that of Srn024 was 2- to 46-fold higher than that of the others (Fig. 1B). This finding confirmed the involvement of CiaRH in the regulation of these csRNAs.

### The direct repeat sequence TTTAAG-N5-TTTAAG is involved in the regulation of Srn024.

**FIG 2:**
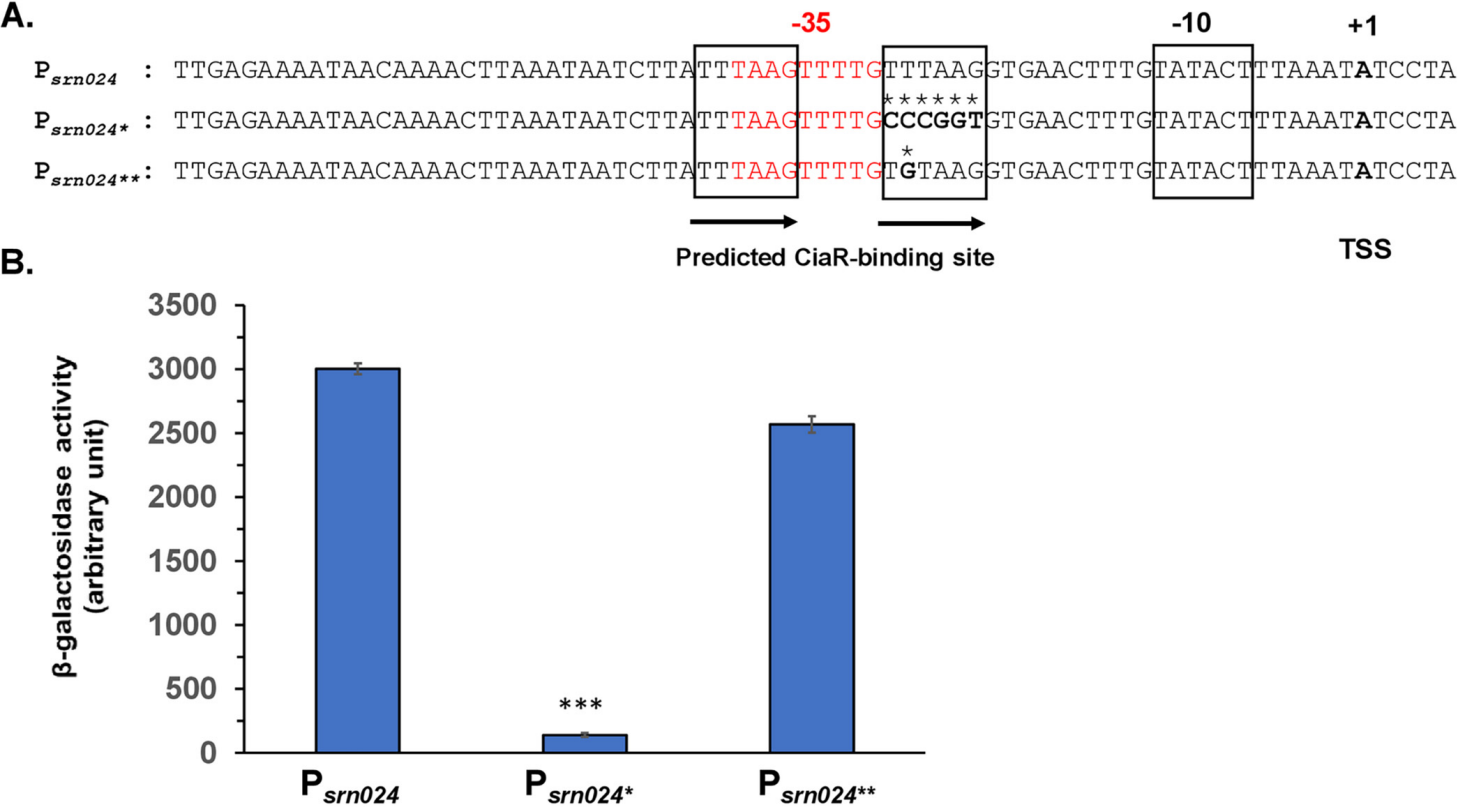
Involvement of the CiaR binding site in the regulation of Srn024. (A) Sequences of native (P*_srn024_*) and mutated (P_srn024__*_ and P_srn024__**_) promoters of the *srn024* gene. The performed substitutions are shown in bold and indicated by stars. In the promoter sequences, the CiaR-related repeat is underlined by arrows and boxed. The transcriptional start site is shown in bold. The putative −35 sequence is shown in red, and the −10 region is boxed. (B) The activity of native and mutated promoters of *srn024* was measured in S. agalactiae strain NEM316. Results are presented as means ± standard deviations of three independent experiments. The significance was determined by ANOVAs and Student *t* tests. ***, *P < *0.001.

To evaluate the role of the predicted CiaR binding site in transcriptional regulation of these csRNAs, point mutations were introduced in the CiaR box of the pTCV-P_s_*_rn024_-lacZ* transcriptional fusion (Fig. 2A); the recombinant plasmids were transformed into the NEM316 strain, and the β-galactosidase activity of the different constructs was measured. Compared to the native promoter (P*_srn024_*), the β-galactosidase activity was strongly reduced when the second TTTAAG motif was replaced by the CCCGGT sequence (P_srn024__*_). However, the β-galactosidase activity was not altered by replacing the second nucleotide of this motif (P_srn024__**_) (Fig. 2). These results confirmed the involvement of the direct repeat sequence TTTAAG-N5-TTTAAG in the transcriptional regulation of Srn024.

### Srn024 does not encode a small peptide.

A putative open reading frame (ORF) predicted in the *srn024* sequence is for a 24-amino acid peptide. Although the sequence is not preceded by a ribosome binding site (RBS), a translational fusion was performed to test the putative synthesis of a peptide. For this, we developed pNJE2, a vector derived from pMSP3545 (see Fig. S1A and B in the supplemental material) (17) containing a nisin-inducible promoter (P*_nisA_*) followed by (i) a transcriptional start site, (ii) a multiple cloning site, and (iii) a transcriptional terminator. Its derivative, pNJE2::*srn024*ORF, contains the putative *srn024* ORF with its upstream sequence cloned in frame and upstream of the *lacZ* gene starting from the second codon (see Fig. S2). No LacZ activity was detected with this construct (see Fig. S2B). Therefore, we conclude that Srn024 likely does not express a peptide; consequently, the phenotypes that we report here are likely due to RNA pairing activities.

### Srn024 targets the LPXTG-cell wall-anchored pullulanase SAP by RNA-RNA pairing *in silico*.

**FIG 3:**
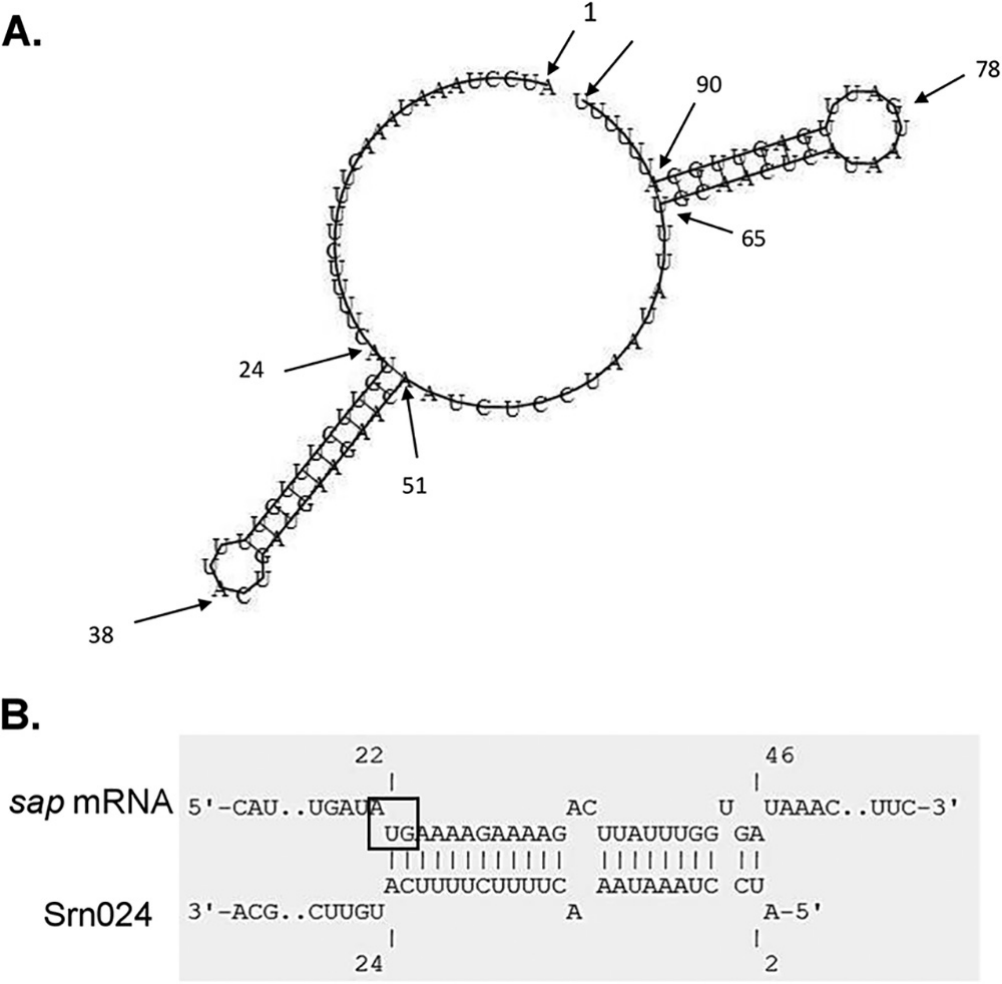
Predicted interaction between Srn024 and *sap* mRNA. (A) Putative secondary structure of the csRNA Srn024, as predicted by RNAfold (43). (B) Putative interaction between csRNA Srn024 and the *sap* mRNA target as predicted by IntaRNA. The first codon of *sap* mRNA is boxed.

To identify putative mRNA targets of Srn024, a genome-wide prediction of Srn024-mRNA interactions was performed using *in silico* target prediction web servers (CopraRNA, TargetRNA2, and RNApredator) (18–20). A list of the top three ranked candidate target genes for Srn024 is shown in Table 1 and described in Table 2. *gbs1288* mRNA, also known as *sap* mRNA (S. agalactiae pullulanase), was the most probable target of Srn024, with the lowest thermodynamic pairing energy between the two RNA molecules for TargetRNA2 (−14.37 kJ/mol) and RNApredator (−17.41 kJ/mol). It was also predicted in third position by CopraRNA (−17.74 kJ/mol) (Table 1). The putative interaction region of 22 nt between Srn024 and the *sap* mRNA predicted by IntaRNA is located between the AUG start codon region of *sap* mRNA and the first 2 to 24 nt of Srn024 (Fig. 3; also see Fig. S3) (18). Thus, *in silico* predictions suggest that Srn024 affects *sap* expression by RNA-RNA pairing.

**TABLE 1:** Top three ranked putative target genes for Srn024 in S. agalactiae strain NEM316

Predicted target rank	Predicted target from CopraRNA (energy [kJ/mol])	Predicted target from TargetRNA2 (energy [kJ/mol])	Predicted target from RNApredator (energy [kJ/mol])
1	*gbs1498* (−18.16)	*gbs1288* (−14.37)^*a*^	*gbs1288* (−17.41)^*a*^
2	*gbs1396* (−18.04)	*gbs1398* (−13.95)	*gbs1396* (−17.20)
3	*gbs1288* (−17.74)^*a*^	*gbs0877* (−13.57)	*gbs0609* (−15.41)

a Gene predicted by the three tools.

**TABLE 2:** Description of putative target genes of Srn024 in S. agalactiae strain NEM316

Gene name	Description	Genomic coordinates^*a*^
*gbs0609*	HAD-IA family hydrolase	633184–631920
*gbs0877* (*atpF*)	ATP synthase subunit B	904415–904912
*gbs1288* (*sap*)	Pullulanase	1333559–1329601
*gbs1396*	Mevalonate kinase	1445104–1444026
*gbs1398*	Response regulator transcription factor	1446935–1446249
*gbs1498* (*mscL*)	Large-conductance mechanosensitive channel	1551434–1551811

a Nucleotides positions on the corresponding genome : begin-end.

### Srn024 does not affect the transcription of *sap*.

To evaluate *sap* transcription, a strain in which the *srn024* gene was inactivated (Δ*srn024*) was constructed along with a complemented strain containing a recombinant vector carrying *srn024* (Δ*srn024*-pTCV-P_Tet_::*srn024*). The amount of *sap* transcripts was determined by reverse transcription-quantitative PCR (RT-qPCR) in NEM316-pTCVP_Tet_ (empty vector), Δ*srn024*-pTCV-P_Tet_, and Δ*srn024*-pTCV-P_Tet_::*srn024* strains. Transcription was not significantly different in the three strains (see Fig. S4). We concluded that the role of Srn024 is not likely to affect *sap* transcription; therefore, Srn024 may act posttranscriptionally.

### Posttranscriptional regulation of *sap* by Srn024.

**FIG 4:**
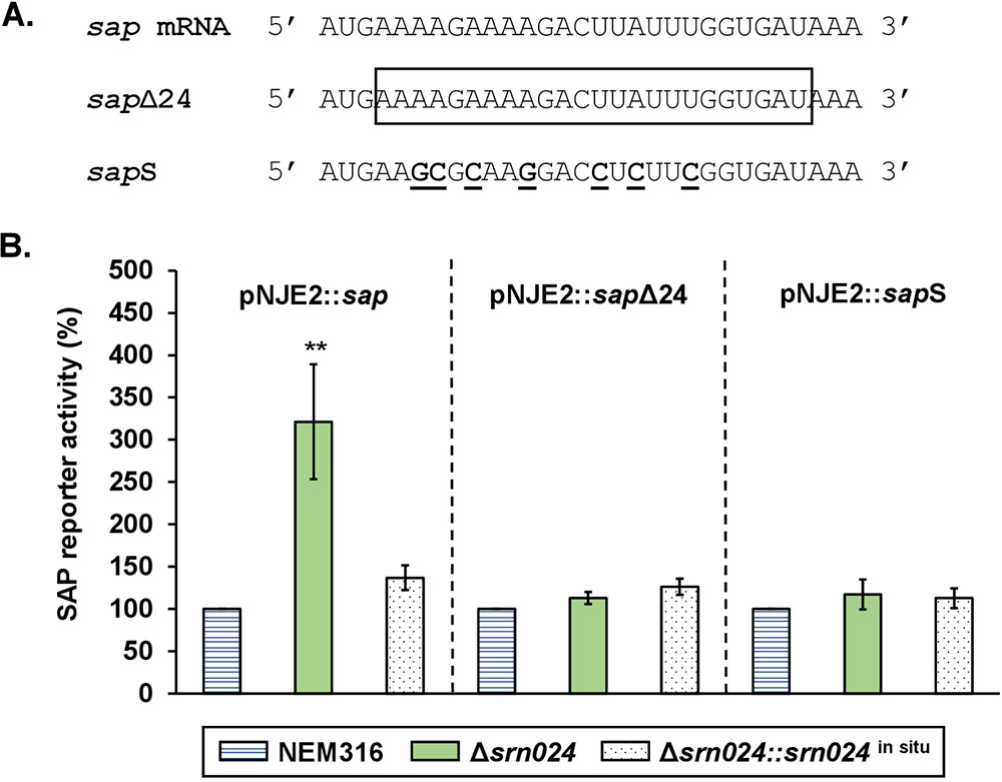
Posttranscriptional regulation of *sap* by Srn024. (A) Sequences of native *sap* mRNA, *sap*Δ*24* (24-nt deletion is boxed), and *sap*S (7-nt substitutions are shown in bold and underlined). (B) Translation of SAP was measured in S. agalactiae NEM316, Δ*Srn024*, and Δ*Srn024*::*Srn024^in situ^* strains. Results are presented as means ± standard deviations of three independent experiments. The significance was determined by ANOVAs and Student *t* tests. **, *P < *0.01.

**FIG 5:**
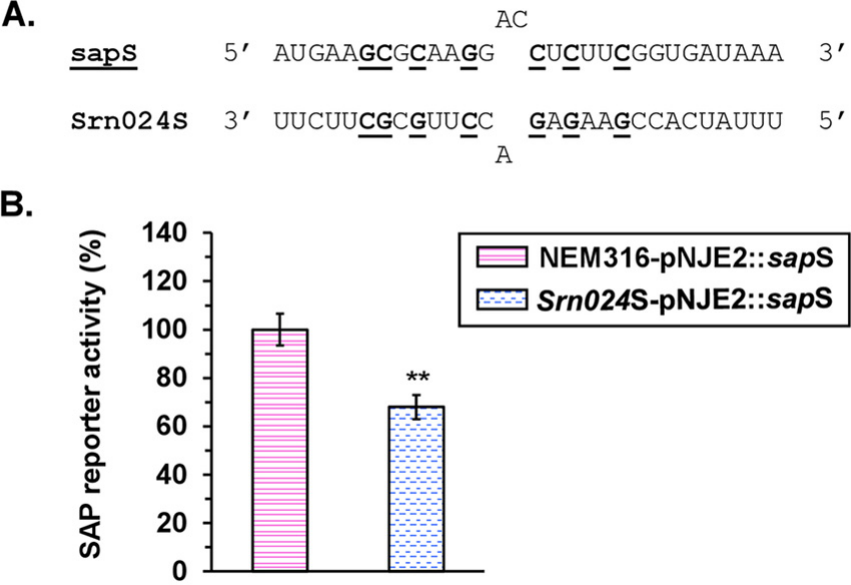
Posttranscriptional regulation of *sap* by Srn024. (A) Putative interaction sequence between *sap* mRNA and Srn024. Performed substitutions are shown in bold and underlined. (B) Translation of SAP was measured in S. agalactiae NEM316 and *Srn024*S strains. Results are presented as means ± standard deviations of three independent experiments. The significance was determined by ANOVAs and Student *t* tests. **, *P < *0.01.

Because *sap* transcription is not altered by Srn024 but is a predicted Srn024 target, we considered that Srn024 could act posttranscriptionally. In order to decipher any involvement of Srn024 in posttranscriptional regulation of *sap* mRNA, we constructed a pNJE2::*sap* vector in which the beginning of the *sap* gene (RBS and translation start codon) was cloned upstream of the *lacZ* gene starting from the second codon (see Fig. S1C). NEM316, Δ*srn024*, and complemented Δ*srn024*::*srn024^in situ^* strains were transformed with the resulting vector pNJE2::*sap*. Expression of SAP-reporter was induced by the addition of nisin (25 ng/mL) to the growth medium. At mid-exponential growth phase, β-galactosidase activity was measured. Compared to Δs*rn024*, the expression of the reporter fusion was about 2- and 3-fold higher in NEM316 and Δ*srn024*::*srn024* strains, respectively (Fig. 4). These results suggest that Srn024 downregulates *sap* mRNA posttranscriptionally, likely by direct sRNA-mRNA interaction. In order to show the involvement of Srn024 in posttranscriptional regulation of *sap* mRNA, pNJE2::*sap* derivative plasmids carrying either a 24-nt deletion (pNJE2::*sap*Δ24) or a 7-nt substitution (pNJE2::*sap*S) in the sequence corresponding to the predicted interaction region of Srn024-*sap* mRNA were introduced into NEM316, Δ*srn024*, and Δ*srn024*::*srn024^in situ^* strains (Fig. 4A). No difference in β-galactosidase activity was observed between the six strains (Fig. 4B). To confirm the importance of this sequence in the regulation of SAP, we constructed a mutant strain carrying 7-nt substitutions in *srn024* gene (*srn024*S) that were complementary to those performed in *sap* (Fig. 5A). SAP-reporter was less expressed in the *srn024*S strain than in NEM316 (Fig. 5B). Together, these experiments appear to support the predicted Srn024-*sap* mRNA interaction.

### Srn024 and CiaRH inhibit S. agalactiae growth in a chemically defined medium and biofilm formation.

**FIG 6:**
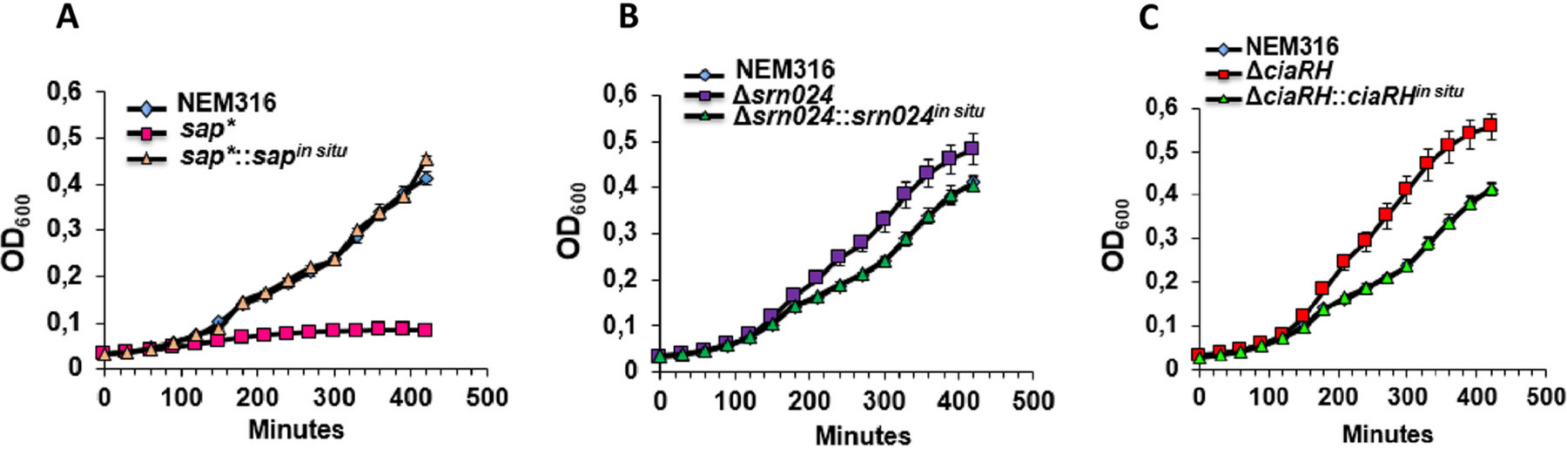
SAP protein is essential for S. agalactiae growth in the presence of pullulan as a unique carbon source, while CiaRH and Srn024 repress its growth in the same medium. Growth of NEM316, sap*, and sap*::*sap^in situ^* strains (A), growth of NEM316, Δ*srn024*, and Δ*srn024*::Δ*srn024^in situ^* strains (B), and growth of NEM316, Δ*ciaRH*, and Δ*ciaRH*::Δ*ciaRH^in situ^* strains (C) in CDM with 1% pullulan was measured. Results are presented as means ± standard deviations of three independent experiments. Significance was determined by ANOVAs and Student *t test*s using the WT strain NEM316 as a reference.

Our findings suggesting the effects of Srn024 on SAP expression led us to investigate the roles of Srn024 in S. agalactiae growth in a medium with only pullulan as a carbon source and in biofilm formation. To test these hypotheses, we constructed a strain in which the *sap* gene was inactivated (sap*). In a chemically defined medium (CDM) with only 1% pullulan as a carbon source, the sap* strain did not grow while the Δ*srn024* and Δ*ciaRH* strains grew faster than the WT strain (Fig. 6).

**FIG 7:**
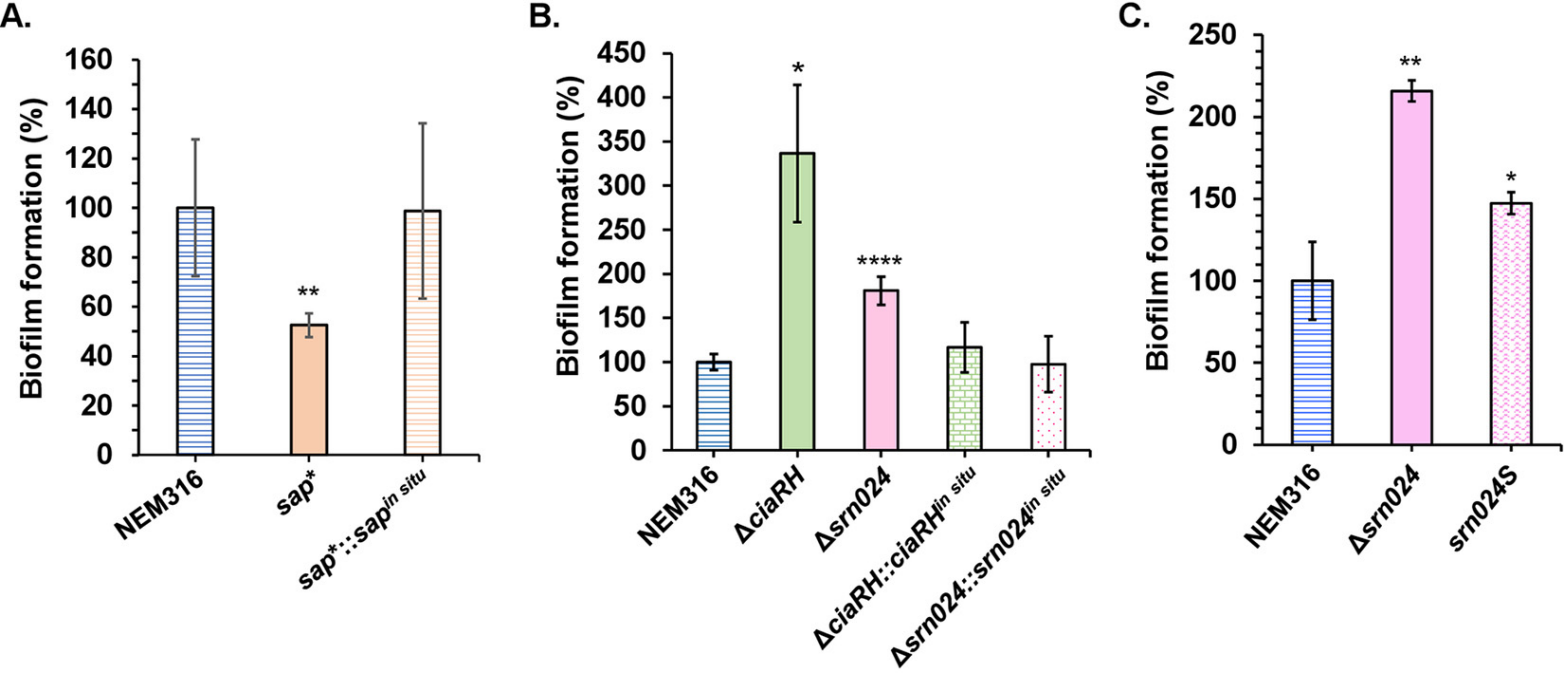
SAP increases biofilm formation, while CiaRH and Srn024 inhibit it. Biofilm formation by NEM316, sap*, and sap*::*sap*^i^*^n situ^* strains (A), NEM316, Δ*srn024*, Δ*srn024*::*Srn024*^i^*^n situ^*, Δ*ciaRH*, and Δ*ciaRH*::*ciaRH^in situ^* strains (B), and NEM316, Δ*srn024*, and *srn024S* strains (C) in TH broth supplemented with 1% glucose was measured. Results are presented as means ± standard deviations of at least three independent experiments. The significance was determined by ANOVAs and Student *t* tests. *, *P* < 0.05; **, *P < *0.01; ****, *P < *0.0001.

Biofilm formation depends on adhesion of microorganisms to each other and to biotic or abiotic surfaces. The NEM316 strain forms biofilms on 96-well plastic plates. The sap* strain showed strongly reduced biofilm formation, which was restored by introducing the *sap^in situ^* allele (Fig. 7A), whereas the strain deficient for Srn024 and CiaRH showed increased biofilm formation, which was also restored by introducing the *srn024^in situ^* and *ciaRH^in situ^* alleles (Fig. 7B). Additionally, biofilm formation by the *srn024S* strain, as the Δs*rn024* strain, increased compared to the WT strain (Fig. 7B and C).

We conclude that Srn024, likely through its interaction with *sap* mRNA, contributes to S. agalactiae adaptation to environmental changes and biofilm formation.

## DISCUSSION

Regulatory sRNAs have been recognized to be essential in the regulation of bacterial genes. Their roles in pathogenesis and adaptation of bacteria to the environment are well established. In streptococci, many sRNAs have been predicted by bioinformatics or experimental screenings as next-generation sequencing (RNA-seq) (21). The expression of some sRNAs (csRNAs) is regulated by the CiaRH TCS conserved in streptococci (22). CiaRH controls many cellular processes, including resistance to the immune system, natural competence, and virulence (23–26). However, with the exception of S. pneumoniae and Streptococcus sanguinis, the function of most of these csRNAs remains unknown. Indeed, in S. pneumoniae, csRNAs are involved in competence, autolysis, and lung infection (7, 8, 27). In S. sanguinis, csRNA1-1 has been confirmed to inhibit biofilm formation (28).

In S. agalactiae NEM316, we previously identified four csRNAs that were bioinformatically predicted (9–13). Here, we showed good conservation (>98% nucleotide identity) of these csRNAs in S. agalactiae. Therefore, these genes are part of the core genome of this species, suggesting an important role for bacterial physiology.

To determine their roles in bacterial adaptation to the host, we first confirmed regulation of Srn015, Srn024, Srn070, and Srn085 csRNAs by the CiaRH TCS. In the promoters driving the expression of each of these four csRNAs, we identified the TTTAAG-N5-TTTAAG sequence, which was previously shown to be essential for CiaR binding in S. pneumoniae (7). Here, we confirmed the upregulation of the four csRNAs by the CiaRH TCS, suggesting the strong dependence of these csRNAs on CiaR (Fig. 1). These results were fully consistent with those for S. pneumoniae (7). We observed an important difference in the promoter activity of the four csRNAs (Fig. 1B). This could be due to sequence variability in csRNAs promoters (see Fig. S5 in the supplemental material). The dependence of the activated Srn024 promoter on CiaR is based on the direct repeat of the TTTAAG hexamer. Indeed, the conversion of the second TTTAAG motif to CCCGGT prevented the activity of the Srn024 promoter. However, the modification of TTTAAG to TGTAAG was not sufficient to impact regulation. The regulation of S. agalactiae csRNAs by the CiaRH TCS strongly suggested a role in the adaptation of the bacterium to its environment.

We then searched for genes that might be regulated by Srn024 in S. agalactiae using target prediction web servers (CopraRNA, TargetRNA2, and RNApredator). Among the different candidate target genes, *sap*, encoding a pullulanase, was the most likely target (Table 1). We demonstrated that Srn024 did not affect the *sap* mRNA transcription (see Fig. S2). However, Srn024 appeared to base pair with the coding sequence of *sap* mRNA (Fig. 3B). Using a translational fusion strategy, we confirmed that Srn024 is involved in *sap* mRNA posttranscriptional regulation. Our results suggest that duplex formation between Srn024 and *sap* mRNA could occlude the start site, resulting in translation inhibition (Fig. 4 and 5).

Bacteria need carbon sources to colonize the host; the most common are α-glucans such as starches and glycogen. Indeed, during periods of high estrogen availability, glycogen is found in large amounts in the vaginal epithelium, while dietary starches are abundant in the human colon (29, 30). SAP pullulanase is a surface-exposed protein that is able to hydrolyze starches and glycogen (14). In this study, we demonstrated the inability of a strain deficient in SAP to grow with only pullulan as a carbon source. However, Δ*ciaRH* and Δ*srn024* strains grow better in the presence of pullulan (Fig. 6). Thus, CiaRH may regulate the expression of SAP pullulanase through Srn024 depending on the availability of carbon sources in the bacterial environment.

Genes homologous to *sap* were found in S. pneumoniae, Streptococcus pyogenes, and Streptococcus suis, named *spuA*, *pulA*, and *apuA*, respectively. They have been proposed as key factors in the metabolic pathway involved in bacterial adaptation to host niches (31–33). SpuA was shown to be necessary for virulence in a mouse lung model of infection and to bind to alveolar type II cell glycogen in the lung, PulA to be responsible for adhesion to human epithelial cells, and ApuA to promote adhesion to porcine epithelium (32–35). SAP was also shown to be necessary for S. agalactiae binding to human cervical epithelial cells (15). We can notice here that pullulanases found in different Streptococcus species play a role in adhesion. Based on these results, we investigated whether CiaRH and Srn024 could play a role in biofilm formation and thus in adhesion by regulating the expression of SAP. We demonstrated that CiaRH and Srn024 downregulate the biofilm formation of S. agalactiae, likely through the interaction between the csRNA and *sap* mRNA (Fig. 7). CiaRH is known to be involved in biofilm formation in several streptococcal species such as S. pyogenes, Streptococcus mutans, and Streptococcus gordonii (22). This study is the first one that shows the involvement of the TCS CiaRH and the csRNA Srn024 in S. agalactiae biofilm formation.

Here, we suggest an adaptation mechanism in which CiaRH may play a role in the posttranscriptional regulation of SAP by Srn024 in response to extracellular stimuli detected by the TCS. When bacteria would be exposed to large amounts of glycogen and starch or would have to adhere to the cell surface, CiaRH would no longer activate Srn024 expression, thus preventing the inhibition of posttranscriptional regulation of SAP. However, the regulation model that we propose here requires further work.

In this study, we confirmed the regulation of the four S. agalactiae csRNAs by the TCS CiaRH. Next, we identified the *sap* gene, which is involved in bacterial adaptation to its different environments, as a target of Srn024 (14). We also demonstrated the implication of CiaRH and Srn024 in biofilm formation of S. agalactiae. Although we have confirmed the posttranscriptional regulation of SAP by Srn024, it would also be important to find other targets and characterize the entire role of Srn024 in S. agalactiae. In addition, characterization of other csRNAs will provide insight into the sRNA-dependent regulatory network of S. agalactiae. This may contribute to the development of drugs targeting regulatory RNAs, reducing the ability of pathogens to adapt to host niches (36).

## MATERIALS AND METHODS

### Plasmids, bacterial strains, and growth conditions.

Serotype III S. agalactiae strain NEM316 was the parental strain used for this study (37). Escherichia coli was used for cloning purposes. All plasmids and bacterial strains used in this study are listed in Table S1 in the supplemental material. S. agalactiae and E. coli strains were routinely grown at 37°C statically in TH broth (Difco) and under agitation in Luria-Bertani (LB) broth (MP Biomedicals), respectively. For translational fusions, S. agalactiae strains were cultured in TH broth with the addition of 25 ng/mL of nisin. For growth kinetics, S. agalactiae strains were grown at 37°C without shaking for 18 h in TH broth. Dilution was performed from these cultures to obtain an optical density at 600 nm (OD_600_) of 0.05 in a CDM (38) supplemented with 1% pullulan (Thermo Fisher Scientific). Growth was studied using 96-well microplates (FALCON, USA). The plates were incubated for 24 h at 37°C without shaking in a spectrophotometer (BioTek Instruments, USA). Every 30 min, the OD_600_ was measured after shaking for 5 s. When necessary, E. coli and S. agalactiae strains were grown with erythromycin (150 μg/mL for E. coli or 10 μg/mL for S. agalactiae).

### RNA preparation.

Total RNA was extracted from mid-exponential-phase cells (OD_600_ of 0.6) by using the phenol/TRIzol method (39). The quality of total RNA was checked on agarose.

### RT-qPCR.

RNA samples were treated with TURBO DNase (Ambion) according to the manufacturer’s instructions. The absence of contaminating genomic DNA was checked by PCR using *recA* primers (all primers used in this study are listed in Table S2 in the supplemental material). Gene selection for normalization, RT-qPCR experiments, and data analyses were performed as described previously (36). The expression levels of the tested genes were normalized using the *recA* gene. Presented data were obtained from duplicate measures of three independent RNA sample extractions.

### Construction of c*iaRH* and *srn024* deletion mutants, *srn024* substitutions mutant, and sap* inactivation mutant.

Mutant strains were constructed by allelic exchanges via a two-step homologous recombination process using pG+host1 derivatives as described (40).

S. agalactiae Δ*ciaRH* is a nonpolar mutant of the NEM316 strain with deletion of the coding sequences of *ciaR* and *ciaH* genes. Upstream and downstream flanking regions of *ciaR*/*ciaH* were PCR amplified. A fusion between these two regions was obtained using the BsaI restriction site. The resulting sequence was amplified and cloned into the EcoRI/BamHI restriction sites of the pG+host1. The recombinant plasmid was electroporated in the NEM316 strain. Allelic exchange was performed as described previously (40). To complement this mutant, the entire coding sequence of *ciaRH* was amplified by PCR and cloned in the pG1+host1, as described above. After allelic exchange, the *in situ* chromosomal complementation of *ciaRH* was confirmed by sequencing.

The same cloning strategy was applied to obtain the Δ*srn024* mutant strain. To complement the Δ*srn024* mutant strain, the entire sequence of *srn024* was PCR amplified and the PCR fragment was cloned into the XbaI/PstI restriction sites of the pTCV-P_Tet_ vector, a derivative of the shuttle vector pTCV that carries a constitutively expressed Gram-positive promoter sequence (40). To construct the *in situ* complement, *srn024* was amplified by PCR and inserted into pG1+host1, using the same strategy as for *ciaRH*.

To construct *srn024*S with substitutions of 7 nt in *srn024*, upstream and downstream flanking regions of *srn024* were PCR amplified. A fusion between the two regions was obtained by using splicing-by-overlap extension PCR. The same strategy was used to construct a SAP inactivation mutant (sap*) by adding a stop codon to the beginning of the *sap* sequence.

After ligation, recombinant plasmids were transformed into E. coli, verified by DNA sequencing of the cloned regions, and then electroporated into the appropriate NEM316 S. agalactiae derivative. For strain constructions, the genomic DNA of mutants was isolated, and the presence of appropriate mutations was checked by sequencing the concerned regions.

### Construction of vectors for transcriptional fusion assays.

The pTCV-*lacZ* vector was used to construct transcriptional fusions between the E. coli *lacZ* reporter gene and upstream regions of the *srn015*, *srn024*, *srn070*, and *srn085* genes (41). The intergenic regions containing the four csRNAs promoters were PCR amplified (11). The corresponding purified DNA fragments were cloned into the BamHI/EcoRI restriction sites of the pTCV-*lacZ* vector. NEM316, Δ*ciaRH*, and Δ*ciaRH*::*ciaRH^in situ^* strains were transformed with these constructions. The same cloning strategy was applied to obtain mutations in the *srn024* promoter. A fusion between the two regions of each construction was obtained by using splicing-by-overlap extension PCR.

### β-Galactosidase transcriptional fusion assays.

For quantification of csRNAs expression, TH broth with 10 μg/mL erythromycin was inoculated to an OD_600_ of 0.05 with an overnight culture. This culture was incubated at 37°C without agitation. Bacteria were harvested at mid-exponential growth phase (OD_600_ of 0.6), and β-galactosidase assays were performed as described previously (38). All experiments were carried out at least three times.

### Construction of the pNJE2 vector for translational fusion.

The nisin-inducible promoter (P*_nisA_*) was amplified from the pMSP3545 vector (17). The *lacZ* gene was amplified from its second codon from the pTCV-*lacZ* vector (41). A fusion between these two fragments was obtained using the NcoI restriction site present in primers. The resulting sequence was amplified and cloned into the BglII/XbaI restriction sites of the pMSP3545 vector.

To construct the pNJE2::*sap* vector, the first 90 nt of *sap* coding sequence were amplified by PCR. To construct pNJE2::*sap*Δ24 and pNJE2::*sap*S vectors, sequences upstream and downstream of the *sap* coding sequence were amplified by PCR (for primers, see Table S2 in the supplemental material). A fusion between the two regions was obtained by using splicing-by-overlap extension PCR. The resulting sequences were cloned into the EagI/PstI restriction sites of the pNJE2 vector. To construct pNJE2::*srn024*ORF, the *srn024* gene was amplified and cloned into the NcoI/PstI restriction sites of the pNJE2 vector.

### β-Galactosidase translational fusion assays.

Bacteria were grown in TH broth supplemented with 25 ng/mL of nisin at 37°C without agitation to the mid-exponential growth phase (OD_600_ of 0.6). The bacteria were harvested, and the β-galactosidase activity was measured as described previously (38). All experiments were carried out at least three times.

### Biofilm formation assay.

Biofilm formation was studied using 96-well polystyrene microplates (Costar, Washington, DC, USA). Dilution was performed from overnight cultures to obtain an OD_600_ of 0.05 in the same medium (TH broth supplemented with 1% glucose). Then, 200 μL of this dilution was dispensed into the microplate and incubated at 37°C for 48 h. After incubation, the culture was removed, and the microplate was washed twice with water. Biofilm was stained for 15 min with 0.2% crystal violet (Merck, Darmstadt, Germany) (110 μL per well) and washed four times with water. After air drying, biofilm was dissolved for 1 h at room temperature with ethanol/acetone (80:20 [vol/vol]). The solution was transferred into a new plate, and the OD_600_ was measured using an Eon thermoregulated spectrophotometer plate reader (BioTek Instruments). The biofilm formation index was calculated as the ratio of the OD_600_ after crystal violet treatment to the OD_600_ before treatment. Biofilm formation was represented as a percentage of that of the WT strain.

### Statistical analyses.

Data are presented as the mean ± standard deviation of at least three independent experiments. Analyses of variance (ANOVAs) and Student *t* tests were used to determine the significance of the differences between means (42).
