# Supplementary material for: Biofilm Formation in Streptococcus agalactiae Is Inhibited by a Small Regulatory RNA Regulated by the Two-Component System CiaRH
Source: Microbiol Spectr. 2022 Aug 18;10(5):e00635-22. doi: 10.1128/spectrum.00635-22 (PMC9603419; doi:10.1128/spectrum.00635-22)
Supplement: Supplemental file 1 — Fig. S1 to S5; Tables S1 and S2. Download spectrum.00635-22-s0001.pdf, PDF file, 1.0 MB [file spectrum.00635-22-s0001.pdf]

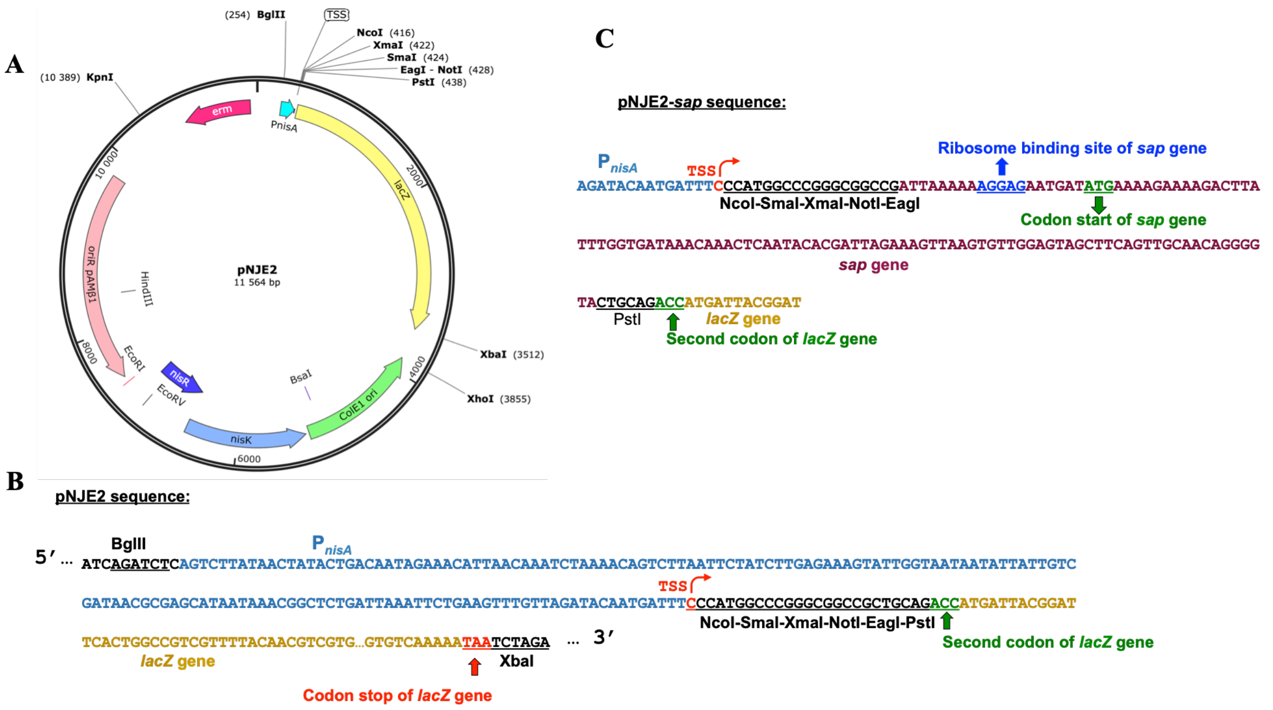

Fig. S1. Schematic representation of (A) pNJE2 translational fusion plasmid organization. (B) pNJE2 sequence between BglII and XbaI restriction sites. (C) The beginning of *sap* gene sequence (ribosome binding site and translation start codon) cloned upstream of the *lacZ* gene in pNJE2 plasmid.

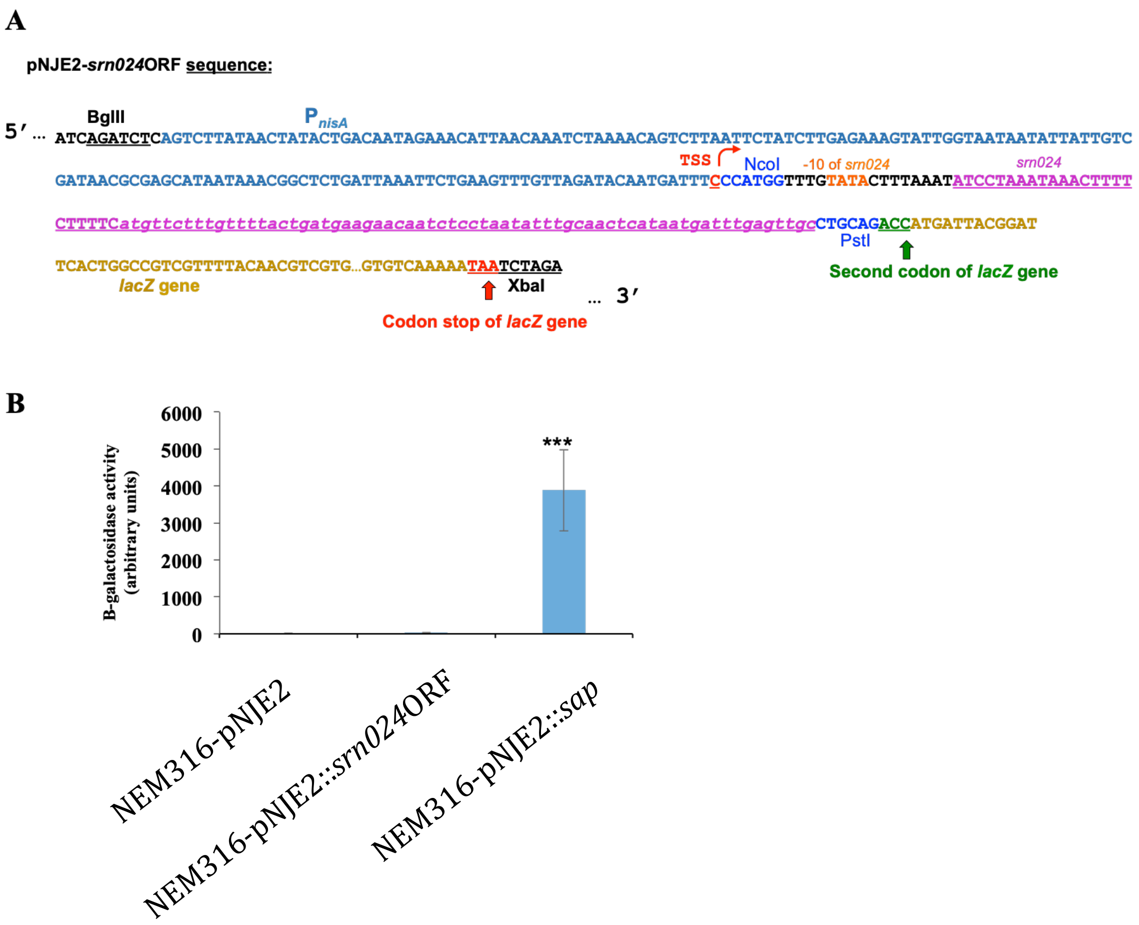

Fig. S2. Srn024 does not encode a small peptide. (A) Sequence of pNJE2::*srn024*ORF. *srn024* sequence is shown in pink and the open reading frame is shown in lower case. (B) β-galactosidase activity was measured in the NEM316-pNJE2 (as negative control), NEM316-pNJE2::*srn024*ORF and NEM316-pNJE2::*sap* (as positive control) strains. Results are presented as means ± standard deviations of three independent experiments. The significance was determined by ANOVAs and Student *t* tests: ***, $P < 0.001$.

```
Srn024

3' ACGUUGAGUUUAGUAAUACUCAACGUUUAUAAUCCUCUAACAAGAAGUAGUCAUUUUGUUUCUUG
                     A
   UACUUUUCUUUUC   AAUAAAUC  CUA  5'

sap mRNA

5' AUGAAAAGAAAAG   UUAUUUGG  GAUAAACAAACUCAAUACACGAUUAGAAAGUUAAGUGUUGGA
                    AC          U
GUAGCUUCAGUUGCAACAGGGGUAUGUAUUUUUCUUCAUAGUCCACAGGUAUUUGCUGAAGAAGUAAGUGUUUCU
CCUGCAACUACAGCGAUUGCAAAGUCGAAUAUUAAUCAGGUUGACAACCGACAAUCUACUAAUUUAAAAGAUGAC
AUAAACUCAAACUCUGAGACGGUUGUGACACCCUCAGAUAUGCCGGAUACCAAGCAAUUAGUAUCAGAUGAAACU
GACACUCAAAAAGGAGUGACAGAGCCGGAUAAGGCGACAAGCCUGCUUGAAGAAAAUAAAGGUCCUGUUUCAGAU
AAAAAUACCUUAGAUUUAAAAGUGGCACCAUCUACAUUGCAAAAUACUCCCGACAAAACUUCUCAAGCUAUAGGU
GCUCCAAGUCCGACCUUGAAAGUUGCUAAUCAAGCUCCACAGAUUGAAAAUGGUUACUUUAGGUUACAUCUUAAA
GAAUUGCCUCAAGGUCAUCCUGUAGAAAGCACUGGGCUUUGGAUAUGGGGAGAUGUUGAUCAACCGUCUAGUAAU
UGGCCAAAUGGUGCUAUCCCUAUGACUAAUGCUAAGAAAGAUGAUUACGGUUAUUAUGUUGAUUUUAAAUUAUCU
GAAAAACAACGAAAACAAAUAUCUUUUUUAAUUAAUAACAAAGCAGGAACAAAUUUAAGCGGCGAUCAUCAUAUU
CCAUUAUUACGACCUGAGAUGAACCAAGUUUGGAUUGAUGAAAAGUACGGUAUACAUACUUAUCAGCCCCUCAAA
GAAGGGUAUGUCCGUAUUAACUAUCUGAGUUCAUCUGGUAACUAUGACCACUUAUCAGCAUGGCUCUUUAAAGAU
GUUGCAACCCCCUCAACAACUUGGCCAGAUGGUAGUAAUUUUGUGAAUCAAGGACUAUAUGGAAGGUAUAUUGAU
GUACCACUGAAAACUAAUGCCAAAGAGAUUGGUUUUCUAAUCUUAGAUGAAAGUAAGACAGGAGAUGCAGUGAAA
GUUCAACCCAACGACUAUGUUUUUAGAGAUUUAGCUAACCAUAACCAAAUUUUUGUAAAAGAUAAGGAUCCAAAG
GUUUAUAAUAAUCCUUAUUACAUUGAUCAAGUGCAGCUAAAGGAUGCUCAACAAACUGAUUUAACAAGUAUUCAA
GCAAGUUUUACAACUCUAGAUGGGGUAGAUAAAACUGAAAUUUUAAAAGAAUUGAAAGUGACAGAUAAAAAUCAA
AAUGCUAUACAAAUUUCUGAUAUCACUCUCGAUACUAGUAAAUCUCUUUUAAUAAUCAAAGGCGACUUUAAUCCU
AAACAAGGUCAUUUCAAUAUAUCUUAUAAUGGUAACAAUGUCACGACAAGGCAAUCUUGGGAAUUUAAAGACCAA
CUUUAUGCUUAUAGUGGAAAUUUAGGUGCAGUUCUCAAUCAAGAUGGUUCAAAAGUUGAAGCCAGCCUCUGGUCA
CCGAGUGCUGAUAGUGUCACUAUGAUUAUUUAUGACAAAGAUAAUCAAAACAGGGUUGUAGCGACUACCCCCCUU
GUGAAAAAUAAUAAAGGUGUUUGGCAGACGAUACUUGAUACUAAAUUAGGUAUUAAAAACUAUACUGGUUACUAU
UAUCUUUACGAAAUAAAAAGAGGUAAGGAUAAGGUUAAGAUUUUAGAUCCUUAUGCAAAGUCAUUAGCAGAGUGG
GAUAGUAAUACUGUUAAUGACGAUAUAAAAACGGCUAAAGCAGCUUUUGUAAAUCCAAGUCAACUUGGACCUCAA
AAUUUAAGUUUUGCUAAAAUUGCUAAUUUUAAAGGAAGACAAGAUGCUGUUAUAUACGAAGCACAUGUAAGAGAC
UUCACUUCUGAUCAAUCUUUGGACGGAAAAUUAAAAAAUCAAUUUGUUACCUUUGCAGCCUUUUCGAGAAACUAG
AUUAUUUACAGAAAUUAGGAGUUACACACAUUCAGCUUUUACCGGUAUUGAGUUAUUUUUAUGUUAAUGAAAUGG
AUAAGUCACGCUCAACAGCUUACACUUCCUCAGACAAUAAUUACAAUUGGGGCUAUGACCCACAGAGCUAUUUUG
CUCUUUCUGGAAUGUAUUCAGAGAAACCAAAAGAUCCAUCAGCACGUAUCGCCGAAUUAAAACAAUUAAUACAUG
AUAUUCAUAAACGUGGCAUGGGGGUUAUACUUGAUGUCGUCUAUAAUCACACUGCAAAAACUUAUCUCUUUGAGG
AUAUAGAACCUAAUUAUUAUCACUUUAUGAAUGAAGAUGGUUCACCAAGAGAAAGUUUUGGAGGGGGACGUUUAG
GAACCACUCAUGCAAUGAGUCGUCGUGUUUUGGUUGAUUCCAUUAAAUAUCUUACAAGUGAAUUUAAAGUUGAUG
GUUUCCGUUUUGAUAUGAUGGGAGAUCAUGAUGCGGCUGCGAUUGAAUUAGCUUAUAAAGAAGCUAAAGCUAUUA
AUCCUAAUAUGAUUAUGAUUGGUGAGGGCUGGAGAACAUUCCAAGGCGAUCAAGGUAAGCCGGUUAAACCAGCUG
ACCAAGAUUGGAUGAAGUCAACCGAUACAGUUGGCGUCUUUUCAGAUGAUAUUCGUAAUAGCUUGAAAUCUGGUU
UUCCAAAUGAAGGUACUCCAGCUUUCAUCACAGGUGGCCCACAAUCUUUACAAGGUAUUUUUAAAAAUAUCAAAG
CACAACCUGGGAAUUUUGAAGCAGAUUCGCCAGGAGAUGUGGUGCAGUAUAUUGCUGCACAUGAUAACCUUACCU
UGCAUGAUGUGAUUGCAAAAUCAAUUAAUAAAGACCCUAAGGUAGCUGAAGAAGAGAUUCAUAGACGUCUGCGUU
UAGGAAAUGUAAUGAUUUUAACAUCUCAAGGGACAGCAUUCAUUCAUUCUGGUCAAGAGUAUGGCCGUACGAAGC
GUUUACUUAACCCUGAUUACAUGACAAAAGUUUCAGAUGACAAAUUGCCUAAUAAAGCAACACUUAUUGAAGCUG
UUAAAGAAUAUCCAUAUUUUAUUCAUGAUUCAUAUGAUUCUUCAGAUGCCAUUAAUCAUUUUGAUUGGGCAGCAG
CCACAGAUAAUAACAAACACCCAAUUUCAACGAAAACACAGGCCUAUACAGCAGGUUUAAUCACAUUAAGGCGUU
CAACAGAUGCUUUCCGGAAAUUGAGCAAAGCAGAAAUUGAUCGUGAGGUUAGCUUGAUUACAGAGGUAGGUCAAG
GUGAUAUUAAAGAAAAAGAUUUGGUUAUUGCUUACCAAACAAUAGAUUCUAAAGGCGAUAUUUACGCAGUAUUUG
UUAAUGCUGAUAGUAAAGCUAGAAACGUUUUACUAGGUGAAAAAUAUAAACACCUUUUAAAAGGGCAAGUAAUUG
UUGAUGCUGAUCAAGCGGGGAUUAAACCAAUCUCAACUCCUAGAGGUGUUCAUUUUGAAAAAGAUAGUUUGCUGA
UUGAUCCAUUAACAGCAAUUGUGAUUAAAGUUGGCAAAGUUGCUCCUAGCCCUAAGGAGGAAUUGCAAGCAGAUU
AUCCCAAAACACAAUCUUUCAAGGGAUCUAAAACGGUAGAAAAAGUAAAUAGAAUAGCUAAUAAGACCUCAAUAA
CUCCUGUAGUUUCUAAUAAGACCGAUUCAUAUCUGACAAAUGAAGCUAAUUUGCCAAAAACUGGAGAUAAGUCAU
CAAAAAUACUAAGUGUAGUAGGAAUAAGCAUUCUAGCAAGUCUACUUGCUCUACUAGGUCUCUCUUUAAAGAGGA
AUCGUCACUUAAAAUAA 3'
```

Fig. S3. Putative interaction between Srn024 and *sap* mRNA target. *sap* mRNA sequence is shown in black. Srn024 sequence is shown in blue. Interaction sequence between Srn024 and *sap* mRNA is shown in red. Translation start codon of *sap* is shown in bold and underlined. Stop translation codon of *sap* is shown in green and underlined.

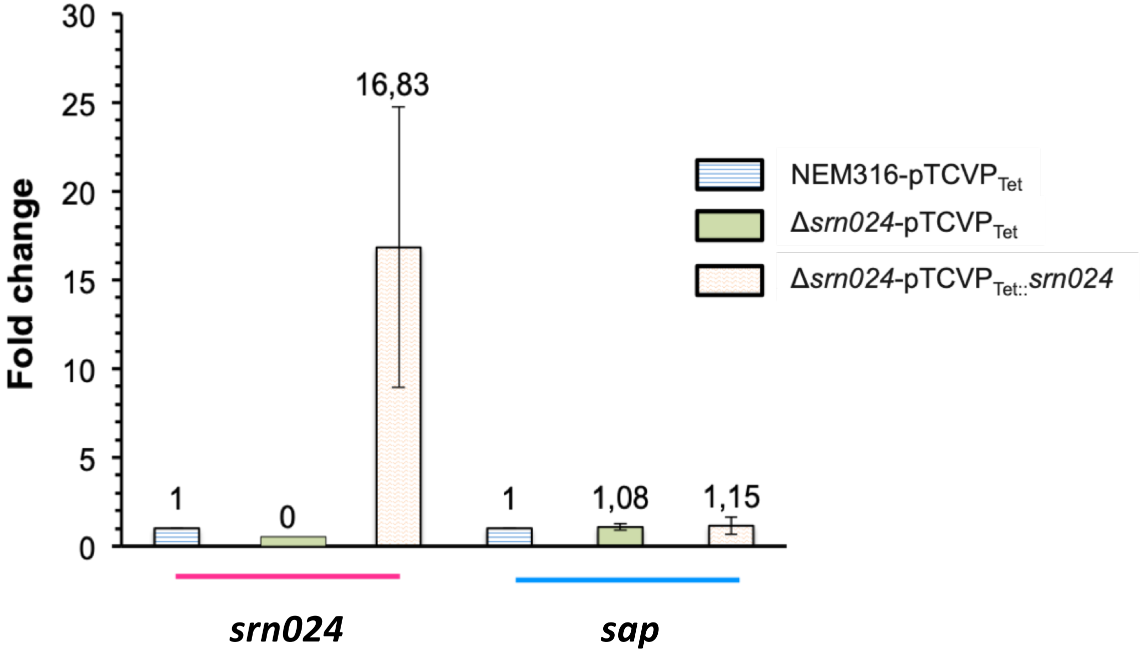

Fig. S4. Srn024 does not affect the transcription of *sap*. Transcription profiling of Srn024 and *sap* in *S. agalactiae* NEM316-pTCV-P$_{Tet}$ (empty vector), Δ*srn024*-pTCV-P$_{Tet}$, and Δ*srn024*-pTCV-P$_{Tet}$::*srn024* strains at mid-exponential growth phase in TH (OD$_{600}$=0.6). Transcription of each gene was normalized to *recA*. Results are presented as means ± standard deviations from three independent experiments.

```
Psrn015                                            -35                      -10        +1
AGGGGAAAATAAAATGATAAAGTATGAATTTTTTAAGGTTTATTTAAGGTTTCTACTGTATACTAGTATCATCCTA

Psrn024
TGAGAAAATAACAAAACTTAAATAATCTTATTTAAGTTTTGTTTAAGGTGAACTTTGTATACTTTAAATATCCTA

Psrn070
ATTTAGGAAGTAATGTTAAATAGCCGAGCTTTTAAGGAAACGTTAAGTAAGGGCGGTTATACTATAAACATCCTA

Psrn085
TCGATGAACTTATTTTACAGAGTAAAACGTTTTAAGACTTGTTTAAGCATGGCCTTATATACTATAACCATCCTA
```

Fig. S5. Sequences of the TCS CiaRH-dependent regulatory RNA promoters in *S. agalactiae*. The putative -35 sequence is shown in green. The -10 sequence is shown in red. The transcription initiation site (+1) is underlined and shown in bold. The potential CiaR binding site is underlined.

Table S1. Strains and plasmids used in this study

Strains or plasmids	Genotype or description	Source or reference
***E. coli* strains**		
**XL1-blue**	*endA1 gyrA96* (Nal^R) *thi-1 recA1 relA1 lac glnV44 hsdR17*(r_K^- m_K^+) F' [::Tn10 (Tet^R) *proAB*$^+$ $lacI^q$ ZΔM15	Stratagene
**BL21 codon +**	F- dcm ompT hsdS (rB - mB -) gal [malB+]K-12 (λS)	Stratagene
***S. agalactiae* strains**		
**NEM316**	Isolated from a case of fatal bacteremia	(1)
ΔciaRH	*ciaRH* deletion mutant of NEM316	This study
**Δ*ciaRH::ciaRH*** $^{\textbf{in situ}}$	*ciaRH in situ* chromosomal complementation of *ΔciaRH*	This study
**Δ*srn024***	*Srn024* deletion mutant of NEM316	This study
**Δ*srn024::srn024*** $^{\textbf{in situ}}$	*srn024 in situ* chromosomal complementation of *Δsrn024*	This study
**Δ*srn024*-pTCVP$_{Tet}$::*srn024***	Plasmid complementation of *Δsrn024*	This study
***srn024*S**	NEM316 with seven nucleotide substitutions in *srn024*	This study
sap*	NEM316 with the mutated sap gene, producing a non-functional protein	This study
**Plasmids**		
**pG+host1^{TS}**	Replication-thermosensitive shuttle vector, Ery^R	(2)
**pTCV-*lacZ***	Promoter probe vector carrying a promotorless l*acZ* gene, Ery^R	(3)

**pTCV-*lacZ::srn015***	pTCV-*lacZ* carrying the *srn015* promotor sequence	This study
**pTCV-*lacZ::srn024***	pTCV-*lacZ* carrying the *srn024* promotor sequence	This study
**pTCV-*lacZ::srn070***	pTCV-*lacZ* carrying the *srn070* promotor sequence	This study
**pTCV-*lacZ::srn085***	pTCV-*lacZ* carrying the *srn085* promotor sequence	This study
**pTCV-*lacZ::srn024****	pTCV-*lacZ* carrying the *srn024* promotor sequence with six mutations in the CiaR binding site	This study
**pTCV-*lacZ::srn024*****	pTCV-*lacZ* carrying the *srn024* promotor sequence with one mutation in the CiaR binding site	This study
**pMSP3545**	Vector carrying a nisin inducible promotor (P*nisA*), Ery^R	(4)
**pNJE2**	Vector carrying a nisin inducible promotor (P*nisA*) and *lacZ* gene devoided of RBS and START codon, Ery^R	This study
**pNJE2::*sap***	pNJE2 carrying the 90 first nucleotides of *sap* coding sequence	This study
**pNJE2::*sap*Δ24**	pNJE2 containing the beginning of *sap* coding sequence with 24 nucleotides deletion	This study
**pNJE2::*sap*S**	pNJE2 containing the beginning of *sap* coding sequence with seven nucleotide mutations	This study
**pNJE2::*srn024*ORF**	pNJE2 containing the beginning of *srn024* gene (with its -10 promoter region) and the potential peptide	This study
**pTCV-P_{tet}**	Mob+(IncP); *oriR pACYC184*; *oriR pAM_1*; complementation vector, promoter P_{Tet}	(5)

**pTCV-P$_{tet}$::*srn024***	*srn024* complementation vector, promoter P$_{Tet}$	This study

REFERENCES RELATED TO SUPPLEMENTAL MATERIAL

1. Glaser P, Rusniok C, Buchrieser C, Chevalier F, Frangeul L, Msadek T, Zouine M, Couvé E, Lalioui L, Poyart C, Trieu-Cuot P, Kunst F. 2002. Genome sequence of *Streptococcus agalactiae*, a pathogen causing invasive neonatal disease. Mol Microbiol 45:1499–1513.
2. Biswas I, Gruss A, Ehrlich SD, Maguin E. 1993. High-efficiency gene inactivation and replacement system for gram-positive bacteria. J Bacteriol 175:3628–3635.
3. Poyart C, Trieu-Cuot P. 1997. A broad-host-range mobilizable shuttle vector for the construction of transcriptional fusions to beta-galactosidase in gram-positive bacteria. FEMS Microbiol Lett 156:193–198.
4. Bryan EM, Bae T, Kleerebezem M, Dunny GM. 2000. Improved Vectors for Nisin-Controlled Expression in Gram-Positive Bacteria. Plasmid 44:183–190.
5. Firon A, Tazi A, Da Cunha V, Brinster S, Sauvage E, Dramsi S, Golenbock DT, Glaser P, Poyart C, Trieu-Cuot P. 2013. The Abi-domain protein Abx1 interacts with the CovS histidine kinase to control virulence gene expression in group B *Streptococcus*. PLoS Pathog 9:e1003179

Table S2. Primers used in this study.

Fonction	oligo	sequence (5'-3')
Amplification of csRNAs promotors	PSrn015-F	ATTCCTGAATTCGTAAGCTTGAGGTTGAGATTTTAAG
	PSrn015-R	ATTTATGGATCCTAGGATAAACCGCTTATTTTAGG
	PSrn024-F	AAACCTGAATTCGTGCTTGATGTACTGAGGAAG
	PSrn024-R	ATTTATGGATCCGAAAAGAAAAGTTTATTTAGGA
	PSrn070-F	ATTGCAGAATTCGAAACCGAGATGTTAAAACTC
	PSrn070-R	ATTCGTGGATCCCAAAATAGAAAAAACACCAAAATAC
	PSrn085-F	ATTGCAGAATTCCTATCGATGAACACCAACA
	PSrn085-R	ATTCGTGGATCCGCAAAAACAAACACCTTG
	PSrn024M-R1	ATACAAAGTTCACACCGGGCAAAACTTAAATAAGATTATTT
	PSrn024M-F2	ATTTAAGTTTTGCCCGGTGTGAACTTTGTATACTTTAAATATCCTA
	PSrn024M1N-R1	GTTCACCTTACACAAAACTTAAATAAGATTATT
	PSrn024M1N-F2	TAAGTTTTGTGTAAGGTGAACTTTGTATACTT
Construction of Δ*ciaRH* and Δ*ciaRH::ciaRH in situ*	CiaR-F	CTAGAATTCCGATGGGAATTTCAAACGAC
	CiaR-R	ATGCTGGGTCTCTGGAAAGACTCAAGTCATCTTCAATT
	CiaH-F	ATGCTGGGTCTCTTTTCCGATAATAAACCTAGAGGCAGT
	CiaH-R	CAGGATCCGGCGCTCTGTTAAACTTTCG
Construction of Δ*srn024* and Δ*srn024* ::*srn024 in situ*	Srn024-F1	TCACTAGGATCCGAGTCGCCTGACACTAGCG
	Srn024-R1	TGGTCTCGTAGGGTCCCCTGCCAACTTCTCTA
	Srn024-F2	TGGTCTCGCCTAATATTTGCAACTCATAATGAT
	Srn024-R2	ATTCCTGAATTCATCCACCAAGATATGCCCGATAA
Construction of Δ*srn024* -pTCVPTet::*srn024*	Srn024-CPF	TCAACTTCTAGAATCCTAAATAAACTTTTCTTTTCATG
	Srn024-CPR	TCAACTCTGCAGAAAAACTTTGAGCAATTTGCTCAAAGTTTTTGCAACTCAAATCATTATGAG

Construction of *srn024* S	Srn024-SCR	ATGAAGCGCAAGGTCTCTTCAGGATATTTAAAGTATACAAAGTTC
	Srn024-SCF	GAAGAGACCTTGCGCTTCATGTTCTTTGTTTTACTGATGAAGAAC
Construction of *sap* *	sap-F1	TCACTAGGATCCGATTATGGTTTTCGTAAGTTAA
	sap-R1	CATTATCCATGGATCATTCTCCTTTTTTAATGAA
	sap-F2	CATATCCCATGGATGAAACAAACTCAATACACGA
	sap-R2	ATTCCTGAATTCTTCTTTAAGATGTAACCTAAAG
Construction of pNJE2	pNJE2-F1	GATACTGCACTATCAACACAC
	pNJE2-R1	CTGCAGCGGCCGCCCGGGCCATGGGAAATCATTGTATCTAACAAACT
	pNJE2-F2	CCCATGGCCCGGGCGGCCGCTGCAGACCATGATTACGGATTCACTG
	pNJE2-R2	GAGCTCTCTAGATTATTTTTGACACCAGACCAACT
Construction of pNJE2::*sap*	sap-F	CATATCCGGCCGATTAAAAAAGGAGAATGATATGAAA
	sap-R	CATATTCTGCAGTACCCCTGTTGCAACTGA
Construction of pNJE2::*sap* Δ24 and pNJE2::*sap* S	sap-FD1	CATATCCGGCCGATTAAAAAAGGAGAATGATATG
	sap-RΔ1	GTATTGAGTTTGTTTCATATCATTCTCCTTTTTTAAT
	sap-FΔ2	AAGGAGAATGATATGAAACAAACTCAATACACGATTA
	sap-RD2	CATATTCTGCAGTTCAGCAAATACCTGTGGAC
	sap-SR	GAAGAGATCCTTGCGCTTCATATCATTCTCCTTTTTTAATGAA
	sap-SF	ATGAAGCGCAAGGACCTCTTCGGTGATAAACAAACTCAATACACG
construction of pNJE2::*srn024* ORF	ORF24-F	TCTACACCATGGTTTGTATACTTTAAATATCCTA

	ORF24-R	TCTACACTGCAGGCAACTCAAATCATTATGAG
qPCR	sap-F	AAGCGGCGATCATCATATTC
	sap-R	ATCTGGCCAAGTTGTTGAGG
	Srn024-F	ATCCTAAATAAACTTTTCTTTTCATGT
	Srn024-R	TTGCAAATATTAGGAGATTGTTCT
	RecA1	TCGCTTTAGGAGCAGGTGGA
	RecA2	GCAGCATAGGCTGGGTCAAG

Restriction sites are underlined
